# Glutamine and partially hydrolyzed guar gum promote intestinal adaptation and modulate small intestinal microbiota in high‐output stoma: A case report

**DOI:** 10.1002/jpr3.70169

**Published:** 2026-03-23

**Authors:** Yunosuke Kawaguchi, Keita Terui, Ayako Takenouchi, Shugo Komatsu, Ryohei Shibata, Katsuhiro Nishimura, Shota Takiguchi, Yukiko Iwase, Rei Hashimoto, Keisuke Matsusaka, Yoshiteru Osone, Jun‐ichiro Ikeda, Tomoro Hishiki

**Affiliations:** ^1^ Department of Pediatric Surgery, Graduate School of Medicine Chiba University Chiba City, Chiba Japan; ^2^ Department of Pediatrics, Graduate School of Medicine Chiba University Chiba 2 Japan; ^3^ Department of Pathology Chiba University Hospital Chiba Japan; ^4^ Department of Diagnostic Pathology, Graduate School of Medicine Chiba University Chiba Japan

**Keywords:** enterocolitis, mucosal, pediatric

## Abstract

We report a case of a very low‐birth‐weight infant with a high‐output stoma following necrotizing enterocolitis. The patient exhibited villous atrophy and microbial dysbiosis. Supplementation with glutamine and partially hydrolyzed guar gum (PHGG) was initiated, leading to reduced stoma output, improved feeding tolerance, and appropriate weight gain. Histological analysis revealed villous elongation, and microbiota analysis showed a shift from *Proteobacteria* dominance to increased *Firmicutes* and *Actinobacteria* abundance, along with increased alpha diversity. These findings suggest that glutamine and PHGG may support intestinal adaptation and microbiota modulation in infants with high‐output stomas. However, conclusions should be drawn cautiously due to the limited generalizability of a single case.

## 1 INTRODUCTION

Glutamine, the most abundant free amino acid in human plasma, is a crucial energy source for intestinal epithelial cells and is essential for maintaining mucosal structure and function.^1^ Glutamine modulates the gut microbiota by promoting the growth of beneficial bacteria and enhancing mucosal immunity by increasing immunoglobulin A secretion.^1^ Partially hydrolyzed guar gum (PHGG), a low‐viscosity soluble dietary fiber derived from guar gum, is readily fermented by the gut microbiota, leading to short‐chain fatty acid production and an improvement in intestinal microbial composition.^2^

Although most studies on the effect of glutamine and PHGG supplementation on gut microbiota in children used stool samples, primarily representing colonic microbiota, the microbiota of the small intestine, the primary site for digestion and nutrient absorption, remains relatively underexplored.^3^ Herein, we describe a pediatric case involving high‐stoma output. We examined the effects of combined glutamine and PHGG administration on intestinal adaptation, mucosal morphology, and small intestinal microbiota composition. The combination was chosen because it is commercially available in Japan and because similar combinations have demonstrated beneficial effects on gut hormone secretion, which may contribute to intestinal villus elongation.

## 2 CASE REPORT

The patient was born at 26 weeks and 3 days of gestation with a birth weight of 421 g. Enteral feeding began on Day 3 of life but was repeatedly discontinued due to elevated inflammatory markers and abdominal distension. On Day 56, the patient started vomiting, and abdominal X‐ray images showed intestinal gas dilatation. The following day (Day 57), the patient was transferred to our hospital for surgical management after unsuccessful conservative treatment. Emergency surgery was performed for necrotizing enterocolitis, a 7‐cm segment of necrotic small intestine was resected, and a double‐barrel enterostomy was created 85 cm from the ligament of Treitz. From postoperative day (POD) 20, enteral feeding (elemental diet formula) was gradually increased. Parenteral nutrition was administered throughout this period and was tapered as enteral feeding was gradually advanced. However, on POD 62, even a small volume of breast milk resulted in a marked increase in stoma output. While stoma output decreased when feeding was stopped, reintroducing even minimal amounts of enteral nutrition consistently led to high‐output stoma losses (>50 g/kg/day).

An enterostomy site biopsy was performed to investigate the etiology of the high‐output stoma. Histological analysis revealed villous atrophy in the small intestine. Therefore, supplementation with Glutamine F (Aido Co.), with glutamine at 0.3 g/kg/day and PHGG at 0.15 g/kg/day, was initiated on POD 112 (Week 0). The glutamine dose (0.3 g/kg/day) was based on previous pediatric clinical trials.^4^ The PHGG dose was determined according to the fixed composition of the commercial formulation. Within 10 days of supplementation, a gradual reduction in stomatal output was observed, and enteral nutrition was increased in a stepwise manner. This was accompanied by appropriate weight gain, and stoma closure was performed on POD 154 (Week 4). The patient was discharged from the hospital on POD 225 without any feeding problems.

**Figure 1:**
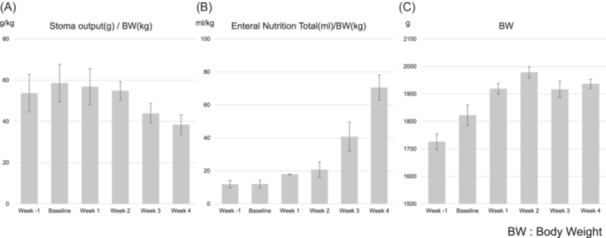
Clinical course following glutamine and PHGG supplementation. (A) Stoma output decreased from 59 ± 8.8 to 38 ± 4.4 g/kg/day by Week 4. (B) Enteral nutrition volume increased from 12 to 70.6 ± 5.2 mL/kg/day by Week 4. (C) Body weight increased from 1822 ± 19 to 1937 ± 22 g by Week 4. Data are presented as mean ± SD. BW, body weight; PHGG, partially hydrolyzed guar gum; SD, standard deviation.

Before glutamine and PHGG supplementation, stoma output averaged 59 ± 8.8 g/kg/day. Four weeks after supplementation, output decreased to 38 ± 4.4 g/kg/day (Figure 1A). Similarly, the enteral feeding volume increased from 12 mL/kg/day before supplementation to 70.6 ± 5.2 mL/kg/day by Week 4 (Figure 1B). Body weight also improved from 1822 ± 19 to 1937 ± 22 g during the same period (Figure 1C).

**Figure 2:**
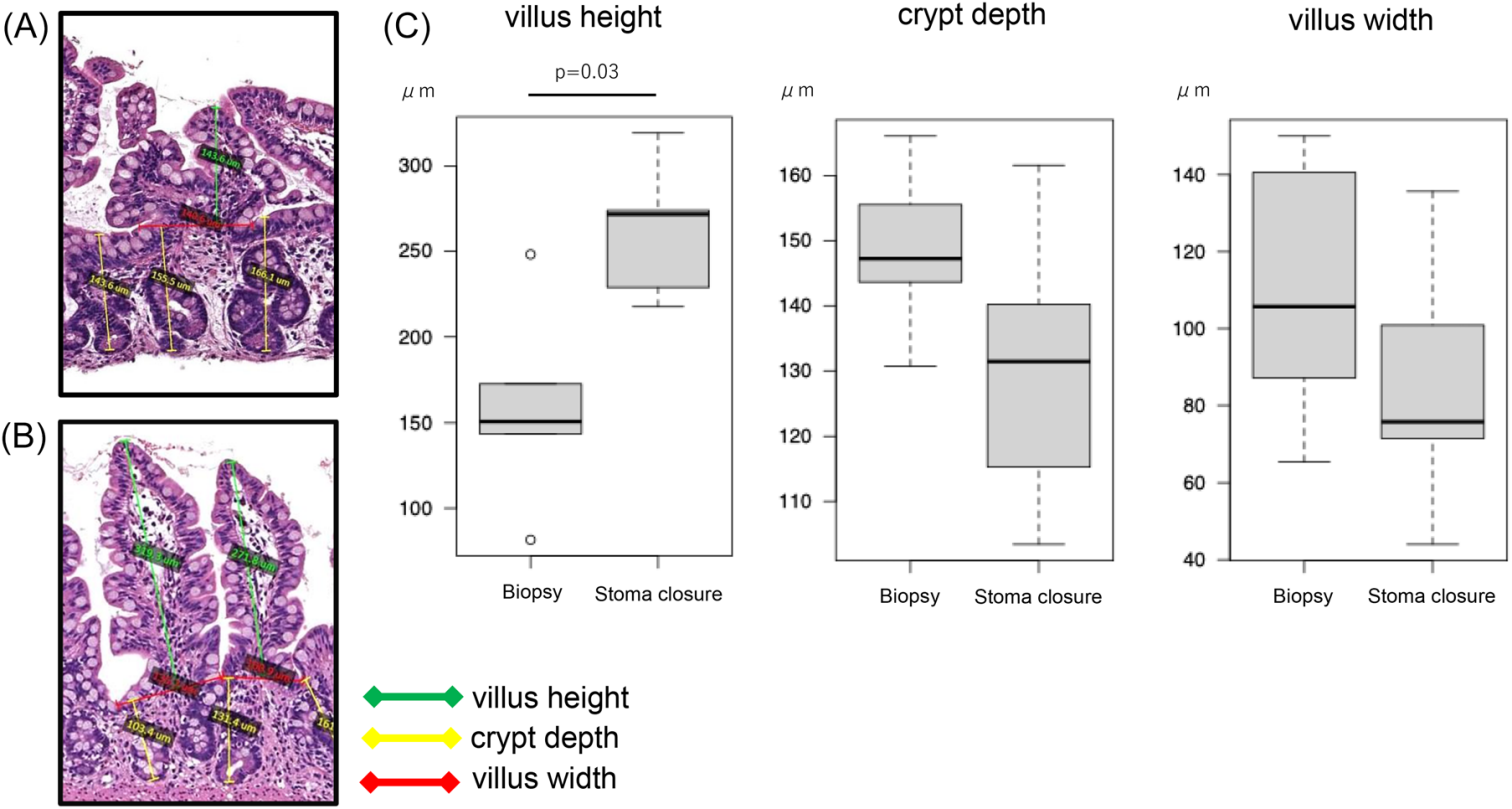
Histological changes in the small intestinal mucosa before and after supplementation. (A) H&E staining of small intestinal biopsy before supplementation (POD 112). (B) H&E staining at the time of stoma closure (POD 154). (C) Quantitative comparison of mucosal parameters. Box‑and‑whisker plots display villus height, crypt depth, and villus width, measured from ≥5 well‑oriented villus–crypt units per specimen. Villus height increased significantly from a median of 144–229 µm (*p* = 0.03, Mann–Whitney *U*‐test). No significant differences were observed in crypt depth (*p* = 0.27) or villus width (*p* = 0.19). Data were analyzed using EZR (ver. 1.73). Green, yellow, and red arrows indicate villus height, crypt depth, and villus width, respectively. H&E, hematoxylin and eosin; POD, postoperative day.

To evaluate changes in small intestinal mucosal morphology, we compared biopsy samples taken from the jejunal mucosa at the enterostomy site before supplementation with those from the resected intestine at stoma closure surgery. We analyzed at least five villus crypt units per sample, as described previously.^5^ Villus height increased from a median of 144 µm at the initial biopsy to 229 µm at stoma closure (*p* = 0.03), whereas crypt depth and villus width did not change significantly (Figure 2).

**Figure 3:**
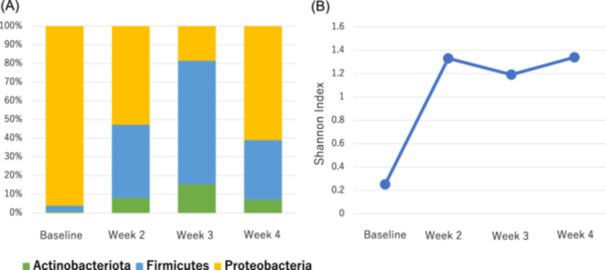
Fecal microbiota changes before and after supplementation. (A) Microbial composition at the phylum level. *Proteobacteria* accounted for 90% before treatment. By Week 2, *Proteobacteria* decreased, whereas *Firmicutes* and *Actinobacteria* increased. (B) Alpha diversity (Shannon index) increased from 0.25 before supplementation to 1.3 at Week 2 and remained stable at Week 4.

The 16s rRNA Amplicon Sequence Microbiota analysis (TechnoSuruga Lab Microbial Identification database) at the phylum level revealed a predominance of *Proteobacteria* (90%) before supplementation. Two weeks after supplementation, the relative abundance of *Proteobacteria* decreased, with a concurrent increase in *Firmicutes* and *Actinobacteria* (Figure 3A). Alpha diversity, assessed using the Shannon index, was initially low (0.25) but increased by Week 2 and remained stable at Week 4, indicating improved dysbiosis following supplementation (Figure 3B).

## 3 DISCUSSION

This case highlights the potential utility of combined glutamine and PHGG supplementation for high‐output stomas in very low‐birth‐weight infants postnecrotizing enterocolitis. The intervention was associated with villous elongation and improved microbial dysbiosis, facilitating enteral feeding and weight gain.

Glutamine, a key energy source for intestinal epithelial cells, helps maintain mucosal homeostasis. When the intestinal tract is not utilized for prolonged periods, such as during parenteral nutrition, intestinal function deteriorates, and the requirement for glutamine increases.^6^ In this case, villous atrophy was observed before the initiation of supplementation, and villous elongation was noted following glutamine and PHGG administration. Although similar effects have been demonstrated in animal models,^7^ this finding suggests that glutamine may also promote mucosal adaptation in infants.

PHGG is a soluble dietary fiber that is fermented by the gut microbiota, resulting in the production of short‐chain fatty acids, including butyrate and acetate, which improve intestinal health. It also enhances stool frequency and consistency and promotes microbial diversity.^8^ In this case, PHGG was associated with reduced stoma output and more viscous stools.

Microbiota analysis showed a shift from *Proteobacteria* dominance to increased *Firmicutes* and *Actinobacteria* populations, alongside improved alpha diversity. Glutamine modulates the microbiota by reducing bacterial overgrowth, translocation, and pathogenic colonization while enhancing secretory immunoglobulin A (IgA) levels and increasing IgA+ plasma cells in the intestinal lamina propria.^9^ PHGG may act synergistically with glutamine, as its prebiotic properties and fermentation‐derived metabolites further support microbial and mucosal health.^8^

Although reports analyzing small intestinal microbiota are limited, previous studies have shown that members of the phylum *Proteobacteria* (e.g., genus *Klebsiella* and *Escherichia–Shigella*) frequently dominate in infants with ileostomies, reflecting microbial dysbiosis.^10^ Interventions such as probiotics (i.e., beneficial bacteria) may also improve outcomes in these patients. As a strength, this case captures changes in the small intestinal environment following nutritional intervention, an area rarely evaluated longitudinally. These results suggest that glutamine and PHGG may represent effective therapeutic options for managing high‐output stomas. However, these findings require caution due to limited generalizability from a single case.

## 4 CONCLUSION

Glutamine and PHGG supplementation may promote mucosal adaptation and improve dysbiosis in infants with high‐output stomas. This case supports their potential as nutritional therapies.

## CONFLICT OF INTEREST STATEMENT

The authors declare no conflicts of interest.

## ETHICS STATEMENT

The study design was approved by the Research Ethics Committee of the Graduate School of Medicine, Chiba University (no. M10168). Written informed consent was obtained from the patient's guardians. The study was performed in accordance with the principles of the Declaration of Helsinki and the Ethical Guidelines for Medical and Health Research Involving Human Subjects.

## Data Availability

Yunosuke Kawaguchi serves as the guarantor of the article and had full access to all data in the study and takes responsibility for the integrity of the data and the decision to submit for publication.
